# Reimbursement of pharmacogenetic tests at a tertiary academic medical center in the United States

**DOI:** 10.3389/fphar.2023.1179364

**Published:** 2023-08-14

**Authors:** Lauren K. Lemke, Benish Alam, Roy Williams, Petr Starostik, Larisa H. Cavallari, Emily J. Cicali, Kristin Wiisanen

**Affiliations:** ^1^ Pharmacotherapy and Translational Research, University of Florida, Gainesville, FL, United States; ^2^ Center for Pharmacogenomics and Precision Medicine, University of Florida, Gainesville, FL, United States; ^3^ Department of Pathology, Immunology and Laboratory Medicine, University of Florida, Gainesville, FL, United States; ^4^ UF Health Pathology Laboratories, UF Health, Gainesville, FL, United States

**Keywords:** pharmacogenetics, pharmacogenomics, insurance coverage, reimbursement, payer

## Abstract

**Introduction:** Pharmacogenetics (PGx) has the potential to improve health outcomes but cost of testing is a barrier for equitable access. Reimbursement by insurance providers may lessen the financial burden for patients, but the extent to which PGx claims are covered in clinical practice has not been well-characterized in the literature.

**Methods:** A retrospective analysis of outpatient claims submitted to payers for PGx tests from 1/1/2019 through 12/31/2021 was performed. A reimbursement rate was calculated and compared across specific test types (e.g., single genes, panel), payers, indication, and the year the claim was submitted.

**Results:** A total of 1,039 outpatient claims for PGx testing were analyzed. The overall reimbursement rate was 46% and ranged from 36%–48% across payers. PGx panels were reimbursed at a significantly higher rate than single gene tests (74% vs. 43%, *p* < 0.001).

**Discussion:** Reimbursement of claims for PGx testing is variable based on the test type, indication, year the claim was submitted, number of diagnosis codes submitted, and number of unique diagnosis codes submitted. Due to the highly variable nature of reimbursement, cost and affordability should be discussed with each patient.

## 1 Introduction

Pharmacogenetics (PGx) involves the use of an individual’s DNA to help predict how they will respond to certain medications. Incorporating PGx into prescribing decisions has been associated with increased medication safety and effectiveness as well as decreased hospital admissions and emergency department visits (Brixner et al., 2016; Reizine et al., 2021; Turongkaravee et al., 2021). There are many gene-drug pairs with evidence to support a change in prescribing; those with the highest quality of evidence are outlined in guidelines published by the Clinical Pharmacogenetics Implementation Consortium (CPIC). To date, there are 26 guidelines published that cover over 140 medications including, but not limited to, CYP2C19-clopidogrel, CYP2D6-opioids, CYP2C19/CYP2D6-selective serotonin reuptake inhibitors (SSRIs), CYP2C19-proton pump inhibitors (PPIs), and TPMT/NUDT15-thiopurines (CPIC, 2023).

Despite the utility of PGx and decreasing cost of genetic testing technology, cost continues to be cited as a major barrier to PGx implementation (Jameson et al., 2021; Gawronski et al., 2023; Wetterstrand, 2023). Patients have a low threshold for out-of-pocket expenses and are more inclined to undergo testing if costs are covered by insurance (Bielinski et al., 2017; Gibson et al., 2017; Liko et al., 2020). The landscape of private payer policies and PGx has been investigated over the last decade with policies indicating coverage for single-gene testing approximately 45% of the time a specific gene test is mentioned in a medical policy (Hresko and Haga, 2012; Graf et al., 2013; Phillips et al., 2017; Lu et al., 2018; Park et al., 2020). For example, one study examined 41 policies and found PGx test were covered 18 of the 56 times testing was mentioned (Hresko and Haga, 2012). However, there are few publications on the reimbursement of PGx tests in practice. A study published in 2020 used claims data for single-gene PGx tests (e.g., *CYP2C19*, *CYP2D6*, *CYP2C9*) submitted to 75 health plans in the United States between 2013 and 2017 to find mean billed and coverage amounts (Anderson et al., 2020). Investigators found all 15,382 PGx tests were at least partially covered through the patients’ insurance, but coverage amounts were declining over the study period.

University of Florida (UF) Health is a large, tertiary academic medical center serving north-central Florida. PGx testing was first piloted in 2011 with *CYP2C19*-clopidogrel and has since expanded to over ten gene-drug pairs in both inpatient and outpatient clinical settings (Johnson et al., 2013; Cavallari et al., 2017). From the initial stages of implementation, PGx testing has been offered by the UF Health Pathology Laboratories. In 2019, a multigene PGx panel was implemented (Marrero et al., 2020). The specific genes offered and the Current Procedural Terminology (CPT) codes billed by the UF Health Pathology lab are outlined in Table 1. Notably, for the data described herein, panels were billed using multiple CPT codes for genes included on the panel *in lieu* of a single, Proprietary Laboratory Analyses (PLA) code.

**TABLE 1:** Available in-house PGx tests and corresponding CPT codes billed.

Single genes		Panel	
Gene	CPT Code Submitted	Genes	CPT Codes Submitted
*CYP2C19*	81225	*CYP2C19*	81225
		*CYP2D6*	81226
*CYP2D6*	81226	*CYP2C9*	81227
		*CYP3A5*	81231
		*SLCO1B1*	81328
*TPMT*	81335	*VKORC1*	81355
		*CYP4F2*	81479
		*CYP2C Cluster*	

Given the paucity of data on recent trends in PGx test coverage and importance of cost on adoption of PGx, we embarked on characterizing trends in reimbursement for PGx tests at a Florida tertiary academic medical center.

## 2 Materials and methods

A retrospective analysis of claims submitted by UF Health Pathology Laboratories to third party payers for outpatient, clinical PGx tests performed between 1 January 2019 and 31 December 2021 was performed. PGx tests funded by research dollars, paid for in cash, performed for inpatients, or performed by a third-party laboratory were excluded.

For each CPT claim, data included date of service, up to three diagnosis codes, payer name, charge, and total net collection. Multiple claims submitted simultaneously for the PGx panel were consolidated and treated as a single claim. CPT codes were translated to the respective PGx test using Codify by the American Academy of Professional Coders, or AAPC (2021). Claims were classified as reimbursed if any amount of reimbursement for the claim was received (i.e., partial or full). Meaning for panels, if only one of the seven CPT codes were reimbursed, it was classified as one paid claim. Reimbursement rate was then calculated by dividing the number of claims reimbursed by the total number of claims submitted. Payer plans were manually sorted into four categories: commercial, Medicare, Medicaid, and other (e.g., TRICARE or VA). Each International Classification of Diseases, 10th Revision (ICD-10) diagnosis code was translated to a text diagnosis using ICD10data.com (ICD, 2021). The first author classified each diagnosis into a broader specialty category (e.g., cardiology); any questionable diagnosis codes were discussed with other authors and classified based on consensus. Herein, use of “diagnosis code” refers to the larger diagnosis category as opposed to a single ICD-10 code. As such, “unique diagnosis code” refers to each unique diagnosis category per claim (e.g., a claim submitted with two cardiology diagnoses and one pain diagnosis would be considered to have two unique diagnosis codes).

The primary endpoint was reimbursement rate, calculated as described above. The reimbursement rate was calculated for the overall data set, for each payer, diagnosis code, PGx test, year of service, number of diagnosis codes, and number of unique diagnosis codes. A subgroup analysis was performed to calculate the reimbursement rate by diagnosis and PGx test as well as payer and PGx test.

Differences between groups of categorical variables were evaluated by the chi-squared test. Subsequent pairwise tests were conducted to determine where any significant differences occur. For pair-wise Chi-squared tests, a Bonferroni adjusted *p*-value was used to control the Family Wise Error Rate. All statistical analysis was conducted in R version 4.1.1 R Core Team (2021).

## 3 Results

**FIGURE 1:**
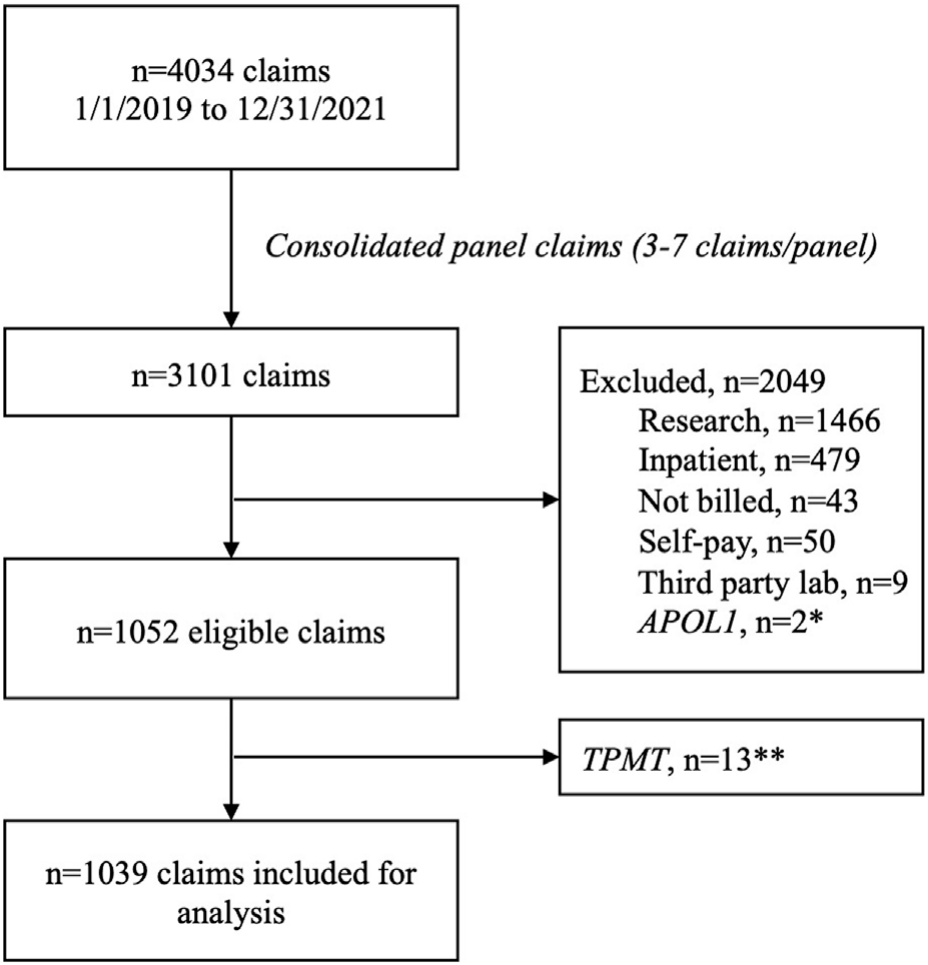
Claims consort diagram. *Excluded due to not being a pharmacogenomics test. **Excluded due to low volume.

Between 1 January 2019 and 31 December 2021 there were 4,034 individual claims submitted by UF Health Pathology Laboratories. After consolidating claims submitted for the panel—which ranged from three to seven claims per panel—to a single claim, 3,101 remained. There were 2,036 claims excluded (including 13 claims for TPMT that were excluded due to low volume) leaving 1,039 claims for analysis (Figure 1). Most claims were submitted for a single gene (89%), and nearly half of the claims were submitted with a psychiatric diagnosis code (49%). Just over half of the claims (53%) were submitted to Medicare or Medicaid and 44% were submitted to commercial payers. The overall reimbursement rate was 46% (Table 2).

**TABLE 2:** Claim reimbursement overview.

Claim characteristic	Total claims submitted, n (%)	Reimbursed n (%)	*p*-value
**All**	1,039 (100)	482 (46)	
**Payer**			
Commercial	460 (44)	222 (48)	0.265
Medicaid	273 (26)	116 (42)	
Medicare	278 (27)	134 (48)	
Other	28 (3)	10 (36)	
**Diagnosis Code**			
Cardiology	281 (27)	147 (52)	0.001
Gastroenterology	203 (20)	90 (44)	
Psychiatry	534 (49)	245 (46)	
Pain	36 (3)	26 (72)	
Other	33 (3)	9 (27)	
**Single Gene**	925 (89)	398 (43)	<0.001
*CYP2C19*	686 (74)	300 (44)	
*CYP2D6*	239 (26)	98 (41)	
**Panel**	114 (11)	84 (74)	
**Year**			
2019	147 (14)	92 (63)	<0.001
2020	249 (24)	106 (43)	
2021	643 (62)	284 (44)	
**Number of Diagnosis Codes**			
1	763 (73)	328 (43)*	<0.001
2	131 (13)	67 (51)	
3	145 (14)	87 (60)*	
**Number of Unique Diagnosis Codes**			
1	900 (87)	397 (44)*	<0.001
2	104 (10)	65 (63)*	
3	35 (3)	20 (57)	

*p < 0.001 based on a pairwise Chi-Square test.

### 3.1 Reimbursement by diagnosis

**FIGURE 2:**
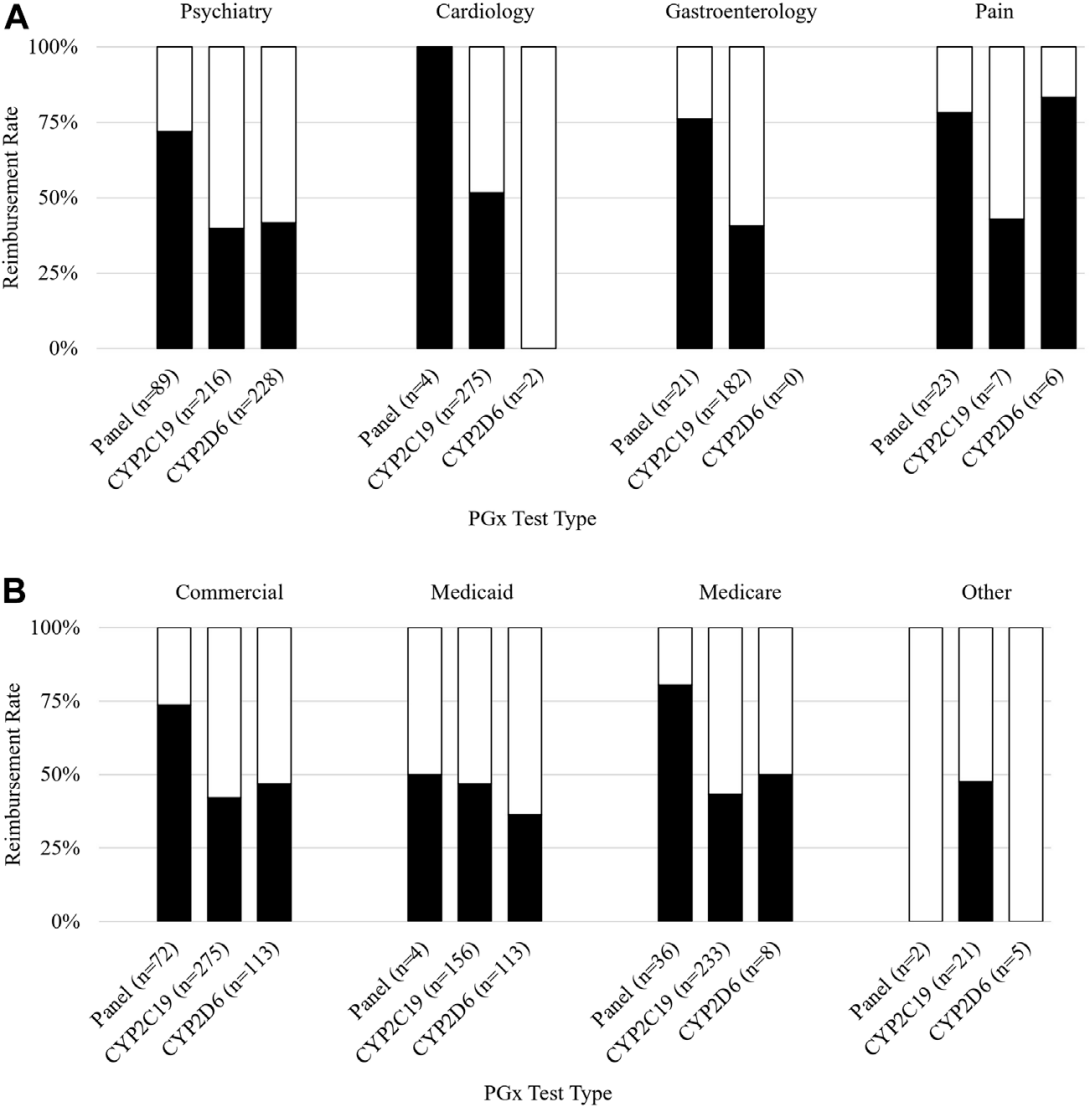
Reimbursement rate subgroup analysis. Figure 2 shows subgroup analysis of reimbursement rate. **(A)** Reimbursement rate (*y*-axis) grouped by payer and further subdivided by PGx test type (*x*-axis). **(B)** Reimbursement rate (*y*-axis) grouped by diagnosis associated with the claim and further subdivided by PGx test type (*x*-axis).

For all claims analyzed, claims for panels (i.e., claims for multiple genes) had a significantly higher reimbursement rate than single-gene tests (74% vs. 43%, *p* < 0.001). Claims submitted with a diagnosis code related to pain were reimbursed most often (72%), followed by claims related to cardiology (52%), psychiatry (46%), gastroenterology (GI) (44%), and other conditions (27%) (*p* = 0.001). A list of specific ICD-10 codes used can be found in Supplementary Table S1. The PGx panel was the test most often performed for an indication of pain (23/36, 64%) followed by the single-gene *CYP2C19* (7/36, 19%) and *CYP2D6* (6/36, 17%) (Figure 2A). The reimbursement rate by test type was 78%, 43%, and 83% for the panel, *CYP2C19*, and *CYP2D6* respectively.

Claims submitted with a diagnosis of cardiology were mostly single-gene *CYP2C19* tests (275/281, 98%), which were reimbursed 52% of the time. Panels and *CYP2D6* each made up 1% of cardiology claims. Cardiology claims for panels (n = 4) were reimbursed 100% of the time. Neither claim for *CYP2D6* (n = 2) was reimbursed.

Almost half of all claims were submitted with a diagnosis relating to psychiatry. The tests performed for the psychiatric claims were most frequently *CYP2D6* (228/534, 43%), *CYP2C19* (216/534, 40%), and panel tests (89/534, 17%); conversely, panels were most frequently reimbursed at a rate of 72% which was significantly higher than *CYP2D6* (42%) (*p* < 0.001) and *CYP2C19* (40%) (*p* < 0.001).

Claims submitted with a GI diagnosis were mostly *CYP2C19* (182/203, 90%), of which 41% were reimbursed. The remaining GI claims were for panels (21/203, 10%) and had a reimbursement rate of 76%.

### 3.2 Reimbursement by payer

An analysis of reimbursement rates by payer and PGx test revealed that panels had higher reimbursement rates than either single gene included on the panel (*CYP2C19* and *CYP2D6*) for commercial payers, Medicaid, and Medicare (Figure 2B). The highest rate of reimbursement for panels was by Medicare and commercial payers (81% and 74%, respectively).

### 3.3 Reimbursement by year

**FIGURE 3:**
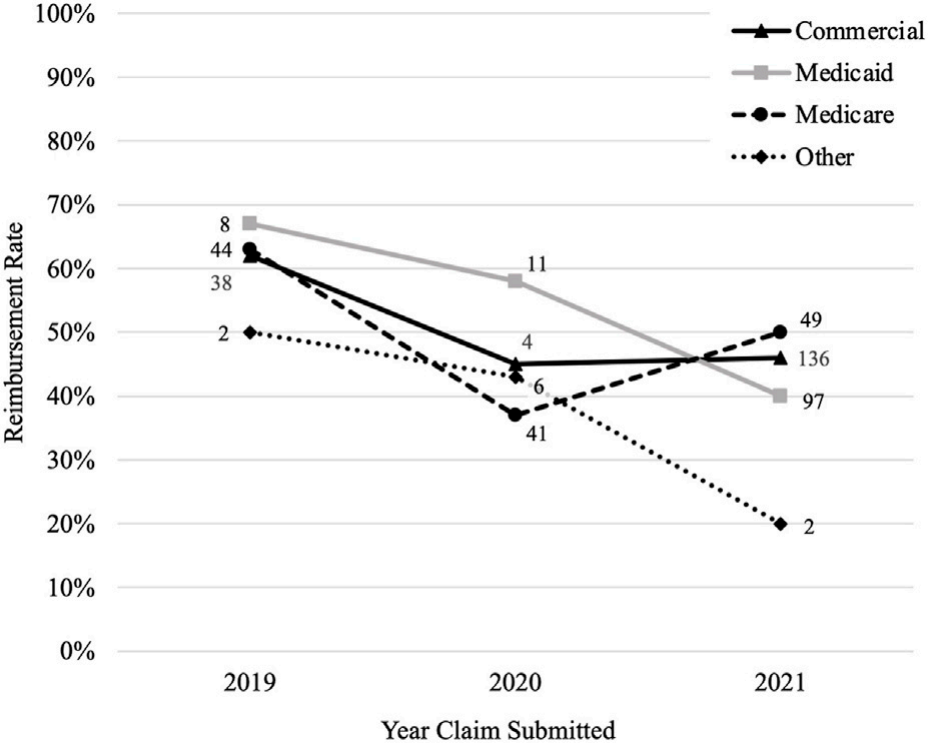
Reimbursement rate by payer overtime. Figure 3 shows how the reimbursement rate for each payer changes over time where the *y*-axis is the reimbursement rate and the *x*-axis is the year the claims were submitted. The number by each data point is the number of claims that were reimbursed by the payer for that year.

Claims submitted in 2019 had the highest reimbursement rate (63%) compared to those submitted in 2020 (43%) and 2021 (44%) (*p* < 0.001). The number of claims increased over time from 147 in 2019, 249 in 2020, and 643 in 2021. Each payer had their highest reimbursement rate in 2019 and all decreased in 2020 (Figure 3). The reimbursement rate for commercial payers and Medicare then increased in 2021 (+1% and +13%, respectively), but Medicaid and other payers continued to trend downward (−18% and −23%, respectively).

### 3.4 Reimbursement by number of diagnosis codes

The reimbursement rate for claims submitted with three diagnosis codes was significantly greater than for claims submitted with a single diagnosis code (60% vs. 43%, *p* < 0.001). There was no significant difference in the reimbursement rate for panels with a single diagnosis (20/25, 80%), two diagnoses (20/30, 67%), or three diagnoses (44/59, 75%) (*p* = 0.49). When comparing the number of unique diagnosis codes submitted with each claim, the reimbursement rate was significantly greater for claims submitted with two unique diagnosis codes than for those with a single diagnosis code (63% vs. 44%, *p* < 0.001). Again, the reimbursement rate for panels did not differ significantly based on the number of unique diagnosis codes used but did trend in support of more unique indications (70% vs. 73% vs. 88%) (*p* = 0.38).

## 4 Discussion

This was a retrospective analysis of 1,039 claims for PGx tests submitted to various payers over 3 years to describe trends in reimbursement of PGx testing. We found an overall reimbursement rate of 46% and a reimbursement rate of 74% for our eight-gene PGx panel. Reimbursement rate was significantly influenced by type of PGx test, diagnosis code, year the claim was submitted, number of diagnosis codes submitted, and number of unique diagnosis codes submitted.

In a review of the largest five private payers’ policies from 2015, Phillips et al. (2017) found multi-gene tests for drug metabolism were mentioned in 25 policies as not covered (Phillips et al., 2017). This is in direct conflict with our findings of commercial payers reimbursing claims for panels 74% of the time. Park et al. (2020) conducted a similar review of policies from 2017 but focused only on single genes addressed in policies from the top 41 private payers in the United States (Park et al., 2020). They found 37 of the 41 payers had at least one policy mentioning PGx, and testing was stated to be covered 46% of all mentioned. In 2012, Hresko and Haga published a similar review of policies from the top ten private payers (Hresko and Haga, 2012). They found half of the companies had at least one policy mentioning PGx, and testing was covered 32% of the time it was mentioned. In their review of claims data, Anderson et al. (2020) found 100% of the 15,382 single-gene PGx tests submitted were at least partially reimbursed, but to a decreasing amount over time (Anderson et al., 2020). Our findings more closely align with Park and Hresko’s, as our claims for single-gene tests were reimbursed 43% of the time. These findings demonstrate the impact time, and potentially policy *versus* practice, has on coverage of PGx tests. This is further supported by our findings that reimbursement rates were significantly associated with the year they were submitted.

On a national scale, the PGx coverage landscape has significantly shifted in the past few years, with major coverage milestones from both commercial and public payers (Empey et al., 2021). In July of 2020, the Medicare Administrative Contractors (MACs) began implementing policies that comprehensively cover PGx testing. The MAC policy applicable to the claims herein did not go into effect until December of 2021, so its impact is minimally reflected in this study. To date, all but one MAC have approved comprehensive coverage for PGx testing, increasing accessibility for over 48 million Medicare beneficiaries (CMS, 2021a; CMS, 2021b; CMS, 2022). Additionally, mandatory coverage for biomarker testing—including markers for “pharmacologic response”—has recently been signed into law in Illinois, Arizona, and Rhode Island (Devino, 2022). The American Cancer Society—Cancer Action Network continues to advocate for expanding coverage at the state legislative level, which will continue to shape reimbursement for PGx testing.

We found reimbursement rate was significantly associated with the diagnoses submitted with each claim. Claims submitted with a diagnosis of pain had the highest rate of reimbursement compared to all other diagnoses. Of these claims, those submitted for the single-gene *CYP2D6* had the highest reimbursement rate at 83%. Panels, which include *CYP2C9* in addition to *CYP2D6*, performed for a pain diagnosis also had a high rate of reimbursement at 78%. This is promising for the use of PGx testing for ambulatory prescribing of opioid analgesics and nonsteroidal anti-inflammatory drugs (NSAIDs), which are impacted by *CYP2D6* and *CYP2C9*, respectively. A minority of claims (7/36) were submitted for *CYP2C19* and had the lowest reimbursement rate (3/7, 43%). Currently, there are no analgesic agents known to significantly interact with *CYP2C19*. Tertiary tricyclic antidepressants (TCAs) may be used for pain, and they rely on CYP2C19 for metabolism; however, genetic variation in *CYP2C19* is not clinically significant at the lower TCA doses used to treat pain (Hicks et al., 2017).

Claims submitted with a cardiology diagnosis had the second highest reimbursement rate and were almost all for the single-gene *CYP2C19* test. Both Park and Hresko reported detailed policy information on *CYP2C19* and clopidogrel, an antiplatelet commonly prescribed for prevention of arterial thrombi, in their publications. Park et al. (2020) found *CYP2C19*-clopidogrel was covered 12 of the 26 times it was mentioned (46%) and Hresko and Haga (2012) found *CYP2C19*-clopidogrel was covered 50% of the four times it was mentioned. This is in line with the reimbursement rate we observed for *CYP2C19* tests submitted with a cardiology diagnosis (52%). In one of the first publications on PGx at UF Health, the reimbursement rate for *CYP2C19* single-gene tests was 85% (Weitzel et al., 2014). The lower reimbursement observed in this data set and stagnation of coverage in payer policies are disappointing given clopidogrel’s Food and Drug Administration (FDA) label has included a Boxed Warning for CYP2C19 poor metabolizers since 2010 (FDA, 2017). The number of claims for panels submitted with a diagnosis related to cardiology was low (n = 4), limiting the conclusions that can be drawn. Continued investigation into the utilization and reimbursement of panels is warranted, as panels may be increasingly ordered for cardiovascular diagnoses to guide prescribing of statins, beta-blockers, and antiarrhythmics in addition to clopidogrel (Duarte and Cavallari, 2021).

Both Park and Hresko also reported detailed policy information on *CYP2C19*-PPIs in their publications, finding testing for *CYP2C19*-PPIs was not covered despite being mentioned 27 times and three times, respectively. We observed *CYP2C19* tests submitted with a GI diagnosis were reimbursed 41% of the time. This difference may be partially explained by increased evidence supporting the impact of *CYP2C19* variation on PPI response evident by the publication of CPIC guidelines for this gene-drug pair in 2020 (Lima et al., 2021).

Claims submitted for a diagnosis related to psychiatry made up almost half of the claims analyzed. SSRIs are first-line agents for the treatment of depression and anxiety and have a CPIC guideline based off their interaction with *CYP2C19* and *CYP2D6* (Hicks et al., 2015; Locke et al., 2015; APA, 2019). Panels, which include both *CYP2C19* and *CYP2D6*, had a significantly higher reimbursement rate than both single-genes, supporting panel-based PGx testing in psychiatry.

Regarding reimbursement rate and the number of diagnosis codes submitted, the percentage of claims reimbursed was highest when more diagnosis codes were submitted per claim. This may reflect medication and indication specific coverage for PGx testing as outlined in commercial payers’ policies (Hresko and Haga, 2012; Park et al., 2020).

There are known inconsistencies between CPIC, the FDA, and other evidence-based sources of PGx information (Shekhani et al., 2020; Shugg et al., 2020; Abdullah-Koolmees et al., 2021; Pritchard et al., 2022). For example, CPIC has guidelines for *CYP2C19*-clopidogrel and *CYP2C19*-PPIs, but the FDA label for clopidogrel has a Boxed Warning for CYP2C19 poor metabolizers and PGx is minimally mentioned in the labels for PPIs. Based on this, one would expect *CYP2C19* testing to be covered more often for clopidogrel as compared to when it is ordered for PPIs, though we observed similar rates of reimbursement for these gene-drug pairs (52% and 41%, respectively).

A significant limitation of this study is the source of the claims data being limited to a single health system representing a small region of the United States. Reassuringly, the characteristics of our claims were similar to that of Anderson et al. in that the most frequent PGx test ordered in both was CYP2C19 (65% of claims) followed by CYP2D6 (26% of claims). Payers were similarly split between Medicare/Medicaid (53% vs. 54%) and commercial (44% vs. 45%). Our dataset was unique in that it included both single-gene and multi-gene PGx tests, where Anderson and others only included single-genes. The similarities of our data to a much larger study performed on a national scale helps support generalizability of our findings. Another limitation lies in the consolidation of multiple single gene claims submitted for a panel. Because panels were made up of three to seven single gene claims, there were 3–7 times more opportunity for reimbursement. This reflects our laboratory’s approach to billing for panels and allows our results to represent our patients’ experience. This also limits the applicability of our results to laboratories that have a single, CPT PLA code for their panel. Additionally, the American Medical Association added a nationally-recognized CPT code for PGx panels in 2023. Furthermore, the timing of our analysis had a 3-week lag from when the last claim was submitted. There is typically a lag time between when a claim is submitted and a coverage decision is made by the payer. Anecdotally, we have found delaying analysis by 1 month after claim submission is adequate to capture final coverage decisions. However, it is possible that coverage decisions are not finalized and may change after this period of time. Another thing that reduces the utility of our findings is that the claims submitted to Medicare were prior to Florida’s MAC comprehensively covering PGx testing, which went into effect on 12/12/2021 (CMS, 2021c). Additionally, our data did not include copays or other charges to the patient, which could impact affordability of testing.

Our findings show promise for the coverage of PGx testing, though there are multiple variables that influence reimbursement. Cost should be discussed with each patient prior to testing and best practice when affordability is an issue would be to investigate coverage on a patient-by-patient basis given the variability. Future studies using claims data are warranted based off the dynamic nature of PGx reimbursement. Ideally, any future investigation would include data from multiple sites or reach patients on a national scale.

## Data Availability

The raw data supporting the conclusions of this article will be made available by the authors, without undue reservation.
