# Supplementary material for: Reimbursement of pharmacogenetic tests at a tertiary academic medical center in the United States
Source: Front Pharmacol. 2023 Aug 14;14:1179364. doi: 10.3389/fphar.2023.1179364 (PMC10461057; doi:10.3389/fphar.2023.1179364)
Supplement: Supplementary file 1 [file Table1.docx]

Supplementary Material

Reimbursement of Pharmacogenetic Tests at a Tertiary Academic Medical Center in the United States

Lauren Lemke^1,2^, Benish Alam^1,2^, Roy Williams^1^, Petr Starostik^3,4^, Larisa Cavallari^1,2^, Emily Cicali^1,2^†, Kristin Wiisanen^1,2^†*

^1^Pharmacotherapy and Translational Research, University of Florida, Gainesville, FL, USA

^2^Center for Pharmacogenomics and Precision Medicine, University of Florida, Gainesville, FL, USA

^3^Department of Pathology, Immunology, and Laboratory Medicine, University of Florida, Gainesville, FL, USA

^4^UF Health Pathology Laboratories, UF Health, Gainesville, FL, USA

†These authors share senior authorship

*** Correspondence:** Kristin Wiisanen: kwiisanen@cop.ufl.edu

# Supplementary Data

## Supplementary Table

| Supplementary Table 1. Use and Reimbursement of ICD-10 Codes | | | |
| --- | --- | --- | --- |
| ICD-10 Code | n Total Claims | n Reimbursed | % Reimbursed |
| C18.9 | 2 | 2 | 100% |
| C64.2 | 2 | 2 | 100% |
| C73 | 2 |  | 0% |
| D46.Z | 2 |  | 0% |
| D47.2 | 1 |  | 0% |
| D57.00 | 1 |  | 0% |
| D89.89 | 1 |  | 0% |
| E03.9 | 3 | 2 | 67% |
| E11.22 | 2 |  | 0% |
| E11.65 | 1 |  | 0% |
| E11.8 | 3 |  | 0% |
| E11.9 | 3 | 3 | 100% |
| E72.12 | 7 | 2 | 29% |
| E78.00 | 1 |  | 0% |
| E78.2 | 12 | 8 | 67% |
| E78.49 | 1 | 1 | 100% |
| E78.5 | 1 | 1 | 100% |
| E88.09 | 1 |  | 0% |
| E88.89 | 1 |  | 0% |
| F09 | 1 | 1 | 100% |
| F11.20 | 2 | 1 | 50% |
| F12.10 | 2 |  | 0% |
| F13.10 | 1 | 1 | 100% |
| F17.200 | 1 | 1 | 100% |
| F19.90 | 1 | 1 | 100% |
| F20.0 | 1 | 1 | 100% |
| F20.81 | 1 | 1 | 100% |
| F20.89 | 3 |  | 0% |
| F23 | 4 |  | 0% |
| F29 | 1 | 1 | 100% |
| F31.0 | 2 | 1 | 50% |
| F31.89 | 3 | 3 | 100% |
| F31.9 | 2 | 2 | 100% |
| F32.0 | 7 | 2 | 29% |
| F32.1 | 27 | 11 | 41% |
| F32.2 | 33 | 15 | 45% |
| F32.3 | 3 | 1 | 33% |
| F32.89 | 50 | 21 | 42% |
| F32.9 | 44 | 29 | 66% |
| F32.A | 2 | 2 | 100% |
| F33.0 | 13 | 5 | 38% |
| F33.1 | 55 | 26 | 47% |
| F33.2 | 62 | 35 | 56% |
| F33.3 | 5 | 2 | 40% |
| F33.40 | 1 | 1 | 100% |
| F33.41 | 5 | 3 | 60% |
| F33.42 | 2 | 1 | 50% |
| F33.9 | 18 | 14 | 78% |
| F34.81 | 15 | 4 | 27% |
| F39 | 2 |  | 0% |
| F40.01 | 3 | 2 | 67% |
| F40.10 | 3 | 1 | 33% |
| F40.9 | 2 | 2 | 100% |
| F41.0 | 3 | 2 | 67% |
| F41.1 | 41 | 31 | 76% |
| F41.8 | 12 | 6 | 50% |
| F41.9 | 68 | 40 | 59% |
| F42.2 | 2 | 1 | 50% |
| F42.8 | 1 |  | 0% |
| F42.9 | 2 | 1 | 50% |
| F43.10 | 29 | 8 | 28% |
| F43.12 | 1 | 1 | 100% |
| F43.20 | 2 |  | 0% |
| F43.23 | 4 | 1 | 25% |
| F43.9 | 11 | 4 | 36% |
| F45.9 | 1 |  | 0% |
| F50.89 | 3 | 1 | 33% |
| F50.9 | 2 |  | 0% |
| F52.32 | 2 |  | 0% |
| F60.3 | 2 | 2 | 100% |
| F63.9 | 3 |  | 0% |
| F84.0 | 2 | 1 | 50% |
| F90.0 | 6 | 2 | 33% |
| F90.2 | 21 | 7 | 33% |
| F90.8 | 1 |  | 0% |
| F90.9 | 13 | 3 | 23% |
| F91.1 | 1 |  | 0% |
| F91.3 | 6 |  | 0% |
| F91.9 | 1 |  | 0% |
| F93.0 | 2 |  | 0% |
| F99 | 2 |  | 0% |
| G45.9 | 2 |  | 0% |
| G47.30 | 2 | 2 | 100% |
| G56.03 | 1 | 1 | 100% |
| G57.93 | 4 | 2 | 50% |
| G62.9 | 4 | 4 | 100% |
| G89.29 | 2 | 2 | 100% |
| G89.4 | 3 | 1 | 33% |
| G90.513 | 1 |  | 0% |
| G93.2 | 1 |  | 0% |
| I10 | 11 | 3 | 27% |
| I15.2 | 2 | 2 | 100% |
| I20.0 | 4 | 2 | 50% |
| I20.9 | 2 | 2 | 100% |
| I21.02 | 10 |  | 0% |
| I21.09 | 1 |  | 0% |
| I21.11 | 19 | 10 | 53% |
| I21.19 | 7 | 3 | 43% |
| I21.21 | 8 | 5 | 63% |
| I21.29 | 7 | 5 | 71% |
| I21.3 | 26 | 21 | 81% |
| I21.4 | 104 | 82 | 79% |
| I21.A9 | 1 | 1 | 100% |
| I24.8 | 1 | 1 | 100% |
| I24.9 | 2 | 1 | 50% |
| I25.10 | 60 | 16 | 27% |
| I25.110 | 2 | 2 | 100% |
| I25.111 | 1 |  | 0% |
| I25.118 | 3 | 3 | 100% |
| I25.119 | 2 | 2 | 100% |
| I25.2 | 1 |  | 0% |
| I25.42 | 1 | 1 | 100% |
| I25.5 | 2 |  | 0% |
| I35.9 | 1 |  | 0% |
| I42.2 | 2 |  | 0% |
| I46.9 | 3 |  | 0% |
| I47.1 | 2 | 2 | 100% |
| I48.1 | 2 | 2 | 100% |
| I50.31 | 1 | 1 | 100% |
| I60.9 | 2 |  | 0% |
| I63.9 | 3 | 1 | 33% |
| I65.21 | 1 |  | 0% |
| I65.22 | 1 |  | 0% |
| I65.23 | 3 | 3 | 100% |
| I67.1 | 9 |  | 0% |
| I67.2 | 1 |  | 0% |
| I71.2 | 2 |  | 0% |
| I72.5 | 2 | 1 | 50% |
| I72.9 | 3 |  | 0% |
| J02.9 | 1 |  | 0% |
| K20.0 | 1 |  | 0% |
| K20.80 | 2 |  | 0% |
| K20.9 | 4 | 2 | 50% |
| K20.90 | 4 |  | 0% |
| K21.0 | 14 | 5 | 36% |
| K21.00 | 8 | 3 | 38% |
| K21.9 | 153 | 68 | 44% |
| K22.10 | 2 | 2 | 100% |
| K22.2 | 4 | 2 | 50% |
| K22.70 | 5 | 1 | 20% |
| K22.710 | 2 |  | 0% |
| K22.711 | 3 | 3 | 100% |
| K22.719 | 2 | 1 | 50% |
| K25.7 | 3 | 2 | 67% |
| K25.9 | 6 | 4 | 67% |
| K26.9 | 1 |  | 0% |
| K27.4 | 1 |  | 0% |
| K29.70 | 4 | 4 | 100% |
| K30 | 2 | 2 | 100% |
| K31.6 | 1 | 1 | 100% |
| K31.84 | 6 | 4 | 67% |
| K31.9 | 2 |  | 0% |
| K44.9 | 5 |  | 0% |
| K50.812 | 1 |  | 0% |
| K52.9 | 2 |  | 0% |
| K58.0 | 1 |  | 0% |
| K58.1 | 2 |  | 0% |
| K59.00 | 2 | 2 | 100% |
| K59.09 | 1 | 1 | 100% |
| K90.0 | 1 |  | 0% |
| L03.116 | 1 | 1 | 100% |
| L40.0 | 2 | 2 | 100% |
| L57.0 | 1 | 1 | 100% |
| L57.8 | 2 | 2 | 100% |
| L85.3 | 2 |  | 0% |
| M17.0 | 4 | 4 | 100% |
| M17.11 | 1 | 1 | 100% |
| M17.12 | 1 | 1 | 100% |
| M19.90 | 2 | 2 | 100% |
| M24.9 | 2 | 2 | 100% |
| M25.50 | 1 | 1 | 100% |
| M25.561 | 2 | 2 | 100% |
| M25.562 | 1 |  | 0% |
| M34.9 | 2 | 2 | 100% |
| M47.816 | 5 | 2 | 40% |
| M50.30 | 2 | 2 | 100% |
| M51.36 | 3 | 1 | 33% |
| M54.41 | 1 | 1 | 100% |
| M54.5 | 2 | 2 | 100% |
| M54.50 | 2 | 2 | 100% |
| M54.6 | 2 | 2 | 100% |
| M54.9 | 1 | 1 | 100% |
| M67.951 | 1 | 1 | 100% |
| M79.605 | 2 | 2 | 100% |
| M79.645 | 1 | 1 | 100% |
| M79.7 | 4 | 2 | 50% |
| M85.80 | 2 |  | 0% |
| N17.9 | 2 | 2 | 100% |
| N18.30 | 2 |  | 0% |
| N60.99 | 1 | 1 | 100% |
| N95.2 | 1 | 1 | 100% |
| R05 | 1 |  | 0% |
| R05.3 | 4 | 2 | 50% |
| R06.02 | 1 |  | 0% |
| R06.6 | 2 | 2 | 100% |
| R06.81 | 4 | 4 | 100% |
| R07.2 | 2 | 2 | 100% |
| R07.89 | 8 | 4 | 50% |
| R07.9 | 9 |  | 0% |
| R10.10 | 1 | 1 | 100% |
| R10.11 | 2 | 2 | 100% |
| R10.12 | 1 |  | 0% |
| R10.13 | 14 | 9 | 64% |
| R10.2 | 1 | 1 | 100% |
| R10.31 | 1 |  | 0% |
| R10.9 | 4 | 1 | 25% |
| R11.0 | 3 | 1 | 33% |
| R11.15 | 1 | 1 | 100% |
| R11.2 | 1 |  | 0% |
| R12 | 3 | 1 | 33% |
| R13.10 | 10 | 2 | 20% |
| R13.14 | 2 | 2 | 100% |
| R14.0 | 7 | 3 | 43% |
| R14.2 | 1 |  | 0% |
| R19.7 | 2 | 2 | 100% |
| R19.8 | 2 | 2 | 100% |
| R30.0 | 1 |  | 0% |
| R41.9 | 2 |  | 0% |
| R45.851 | 4 | 1 | 25% |
| R45.89 | 1 | 1 | 100% |
| R56.9 | 2 | 2 | 100% |
| R59.0 | 1 |  | 0% |
| R73.03 | 1 | 1 | 100% |
| R94.31 | 3 | 1 | 33% |
| T14.91XA | 3 | 2 | 67% |
| T18.9XXA | 1 |  | 0% |
| T39.1X1A | 2 |  | 0% |
| T39.1X2A | 3 | 1 | 33% |
| T43.225A | 1 |  | 0% |
| T43.225D | 2 | 2 | 100% |
| T50.905A | 1 |  | 0% |
| T65.91XA | 4 | 4 | 100% |
| T65.92XA | 1 | 1 | 100% |
| T78.40XD | 1 | 1 | 100% |
| T82.855A | 2 | 2 | 100% |
| T82.867A | 1 |  | 0% |
| T82.867D | 2 |  | 0% |
| U07.1 | 1 | 1 | 100% |
| Z00.00 | 1 |  | 0% |
| Z00.129 | 5 | 2 | 40% |
| Z00.8 | 1 |  | 0% |
| Z01.818 | 5 | 2 | 40% |
| Z11.59 | 1 |  | 0% |
| Z13.79 | 62 | 37 | 60% |
| Z51.81 | 15 | 7 | 47% |
| Z79.899 | 7 | 5 | 71% |
| Z86.19 | 3 | 3 | 100% |
| Z87.19 | 5 | 3 | 60% |
| Z88.9 | 2 |  | 0% |
| Z91.89 | 6 |  | 0% |
| Z95.0 | 1 | 1 | 100% |
| Z95.1 | 3 | 1 | 33% |
| Z95.5 | 5 | 2 | 40% |
| Z98.61 | 33 | 7 | 21% |
| Z98.62 | 1 |  | 0% |
| Z98.84 | 2 | 1 | 50% |
| **Total** | **1592** | **791** |  |
